# How to do (or not to do)… Measuring health worker motivation in surveys in low- and middle-income countries

**DOI:** 10.1093/heapol/czx153

**Published:** 2017-11-20

**Authors:** J Borghi, J Lohmann, E Dale, F Meheus, J Goudge, K Oboirien, A Kuwawenaruwa

**Affiliations:** 1 Department of Global Health and Development, London School of Hygiene & Tropical Medicine, 15-17 Tavistock Place, London, UK; 2 Institute of Public Health, Heidelberg University, Heidelberg, Germany; 3 Department of Health Systems Financing, World Health Organization, Geneva, Switzerland; 4 International Agency for Research on Cancer, World Health Organization, Lyon, France; 5 Centre for Health Policy, Wits University, Johannesburg, South Africa; 6 Ifakara Health Institute, Dar es Salaam, Tanzania

**Keywords:** Motivation, health worker, measurement scale, analysis

## Abstract

A health system’s ability to deliver quality health care depends on the availability of motivated health workers, which are insufficient in many low income settings. Increasing policy and researcher attention is directed towards understanding what drives health worker motivation and how different policy interventions affect motivation, as motivation is key to performance and quality of care outcomes. As a result, there is growing interest among researchers in measuring motivation within health worker surveys. However, there is currently limited guidance on how to conceptualize and approach measurement and how to validate or analyse motivation data collected from health worker surveys, resulting in inconsistent and sometimes poor quality measures. This paper begins by discussing how motivation can be conceptualized, then sets out the steps in developing questions to measure motivation within health worker surveys and in ensuring data quality through validity and reliability tests. The paper also discusses analysis of the resulting motivation measure/s. This paper aims to promote high quality research that will generate policy relevant and useful evidence.

Key Messages

- A clear conceptualization of motivation is required prior to measurement.
- When measuring motivation in a new context, formative research and pre-testing is recommended to identify relevant dimensions and formulate items in local language.
- Validation of motivation measures through factor analysis is important. Where motivation dimensions are well known there is potential for greater use of confirmatory factor analysis.

## Introduction

A health system’s ability to deliver quality health care depends on the availability of motivated health workers, which are lacking in many low income settings (Global Health Workforce Alliance 2014). Motivation has been defined as the level of effort and desire to perform well and is an important determinant of quality of care (World Health Organization 2006). Motivation has been associated with lower levels of staff turnover (Bonenberger *et al.* 2014), higher retention, less job burnout and increased performance (Deci *et al.* 2017), including higher quality of care (Alhassan *et al.* 2013).

An increasing number of interventions (Willis-Shattuck *et al.* 2008; Chopra *et al.* 2008) are designed to improve the retention of health workers and promote better service delivery by enhancing their motivation (Alhassan *et al.* 2013). Such interventions include financial incentives, which can be tied to performance targets (P4P) (Engineer et al. 2016), or non-financial incentives such as career development opportunities and training (Agyepong *et al.* 2004), upgrading facility infrastructure, resource availability (Manongi *et al.* 2006), strategies to improve clinical governance through supportive supervision (Bailey *et al.* 2016), audit or quality management processes (Bakker *et al.* 2011). Programme evaluators may want to assess the effect of national reforms or local programmes on health worker motivation (Huillery and Seban 2014; Källander *et al.* 2015; Chin-Quee *et al.* 2016). Health worker motivation studies may also be of interest in their own right to shed light on what drives motivation (Bhatnagar *et al.* 2017; Mbindyo *et al.* 2009a) and help identify which strategies would be most effective in increasing motivation.

The desire to quantify changes in motivation and to understand motivation drivers in part explains the substantial growth in published research reporting results from health worker surveys that measure motivation (e.g. Bonenberger *et al.* 2014; Hotchkiss *et al.* 2015; Mbindyo *et al.* 2009 b; Weldegebriel *et al.* 2016). However, the measurement and analysis of motivation are not straight forward as motivation is not directly observable (Pinder 2008). A vast body of empirical literature has examined work motivation and the factors driving motivation (Pinder 2008), which have been shown to have predictive value in relation to determining health worker effort and performance (Bandura 1982). For public health researchers with no specialist background in psychology or behavioural economics, however, this literature can be daunting. The lack of guidance on the conceptualization and measurement of motivation in health workers, in particular, has resulted in inconsistent and sometimes poor quality measures within the empirical literature. Our paper aims to serve as an entry point and step-by-step guide for public health researchers new to the field and seeking to measure motivation with measurement scales within surveys. This guide can equally be applied to the measurement of related constructs (e.g. satisfaction, attitudes, perceptions) and with populations other than health workers.

This paper begins by discussing how motivation can be conceptualized, then sets out the steps in developing questions to measure motivation within health worker surveys and in ensuring data quality. The paper also discusses analysis of the resulting motivation measure/s. This paper aims to promote high quality and policy relevant research evidence.

### Step 1: conceptualizing motivation

Motivation is a complex construct as indicated in this definition:

‘Work motivation is a set of energetic forces that originate both within as well as beyond an individual’s being, to initiate work-related behaviour, and to determine its form, direction, intensity, and duration’ (Pinder 2008).

A list of the most prominent motivation theories is provided in Box 1. Motivation is usually either conceptualized as a unidimensional construct, where the focus is on the overall quantity of motivation available to drive behaviour (Gow *et al.* 2013; Hagopian *et al.* 2009); or it is conceptualized as a multidimensional construct, with an additional focus, for example, on the composition of qualitatively different types of motivation such as intrinsic and extrinsic motivation (Lohmann *et al.* 2016). For definitions of key terms such as ‘construct’ please refer to Box 2. In some cases, researchers may wish to capture multiple conceptualizations. The choice of approach depends on the research question and, in the case of programme evaluation, one’s theory about how a given programme will affect motivation.

**Table:** Box 1. Overview of motivation theories

Theory	Original source	Brief description of the theory
**Content theories**		
The Need Hierarchy	Maslow, 1943	Maslow's hierarchy of needs is often portrayed in the shape of a pyramid where physiological needs are at the bottom of the pyramid and considered to be most fundamental. These are followed by safety, then love and belonging, which are followed by esteem and finally the need for self-actualization at the top. When applied to work motivation, it implies that physiological needs (such as salary, secure place to work) should be satisfied before anything else.
ERG Theory: Existence, Relatedness, Growth	Alderfer 1972	Developed out of the Maslow’s hierarchy of needs. Existence relates to a person's physical needs such as food, clothing, and shelter, similar to Malsow’s physiological and safety needs. Relatedness is concerned with the desire people have for maintaining important interpersonal relationships. Growth relates to a person's needs of personal development. Unlike Maslow’s theory, lower level need does not necessarily have to be gratified for a higher level to become relevant. This implies that in a workplace managers must recognize their employees’ multiple simultaneous needs.
Two-Factor Theory: Motivators vs. Hygiene Factors	Hertzberg 1959	Basic idea is that factors which lead to satisfaction such as achievement, intrinsic interest in the work, and involvement in decision making, are distinct from those which lead to job dissatisfaction, such as working conditions, salary, and administrative practices.
Learned Need Theory: Need for Achievement, Need for Power, and Need for Affiliation	McClelland 1976	According to McClelland, all humans have three motivators: a need for achievement, a need for affiliation, and a need for power. However, there is one dominant motivator, which is acquired (‘learned’) through life experience and culture. People with different dominant motivators have different characteristics appropriate for different types of job and positions.
**Process theories**		
Equity Theory	Adams 1963	Focuses on outcomes, a person’s perception of fairness as a motivator. It introduced the concept of social comparison where motivation is based on what a person considers to be fair when compared to others. Employees who perceive inequity when comparing themselves to others in the organization will seek to eliminate it by altering inputs or outputs.
Expectancy Theory: Job Outcomes, Valences, Instrumentality, and Expectancy	Vroom 1964	Defined as an action-outcome estimate: people choose their behaviors (effort level) based on their perceptions of whether the behavior is likely to lead to valued outcomes.
Reinforcement Theory or Operant Conditioning: Stimulus, Response and Consequence	Skinner 1969	Behavior as a ‘function of its consequences,’ desirable behavior can be increased through rewards or reinforcement techniques. Reinforcers can be financial or non-financial (i.e. informational).
Cognitive Evaluation Theory (CET): Intrinsic and Extrinsic Motivation	Porter and Lawler 1968; deCharms 1968	Building on Vroom’s (1964) theory of motivation, Porter and Lawler (1968) proposed a model of intrinsic and extrinsic work motivation, where it appeared that contingent, tangible rewards and other extrinsic factors such as competition and evaluations could undermine intrinsic motivation. Basic assumption in CET is that people have an innate need to feel autonomous and competent, and contingent rewards could undermine these feelings.
Goal Setting Theory	Locke and Latham 1984	People’s actions are driven by goals, they exert more effort when they have specific goals which are difficult but are seen as attainable. Goals need to be accepted, hence the importance of the goal setting process.
Social Cognitive Theory (self-efficacy)	Bandura 1977	Belief in one’s capabilities to successfully execute the behavior, which is needed for a particular task. Experiments showed that even holding abilities constant, people who were more confident exerted more effort, persisted longer and performed better at a task than those who had less confidence.
Self-Determination Theory	Deci and Ryan 2002	Expands on CET, moving away from a simple dichotomy of intrinsic vs. extrinsic motivation. It characterizes extrinsic motivation as a continuum where there are many ‘types’ of extrinsic motivation which differ in their degree of autonomy and internalization. Between amotivation and intrinsic motivation, along a continuum, there are four types of extrinsic motivation, with external being the most controlled type of extrinsic motivation, and introjected, identified, and integrated being progressively more self-determined or autonomous.

Sources: Dale 2014; Shortell and Kaluzny 2006; Mitchell 1997.

Box 2. Defining concepts

**Construct:** used to refer to motivation as a theoretical concept.

**Dimension**: Dimensions refer to sub categories of motivation when motivation is conceptualized as being multi-dimensional. For example, extrinsic and intrinsic motivation may be identified as distinct dimensions of motivation. Dimensions can be predefined based on theory and literature, and/or emerge during the research process, where there is no clear understanding of the construct in a specific context.

**Factor:** The term ‘factor’ used in factor analysis language to refer to the unobservable (or latent) dimension in question, which is measured by the items pertaining to it. For instance, the unobservable factor ‘intrinsic motivation’ might be indicated/measured by five directly observable items (in the sense that respondents give observable answers to these items).

**Item:** refers to a statement or question in a survey tool to measure motivation.

**Response scale:** refers to the response options presented to the respondent in relation to an item (survey statement/question). The term is used especially when there are multiple, ordered response options such as with a Likert scale.

**Measurement scale (or just ‘scale’):** refers to a set of items intended to measure the same construct (e.g. motivation)

**Survey:** refers to the entire questionnaire which usually includes more than one measurement scale and a variety of other questions (demographic, work related).

**Figure 1.**
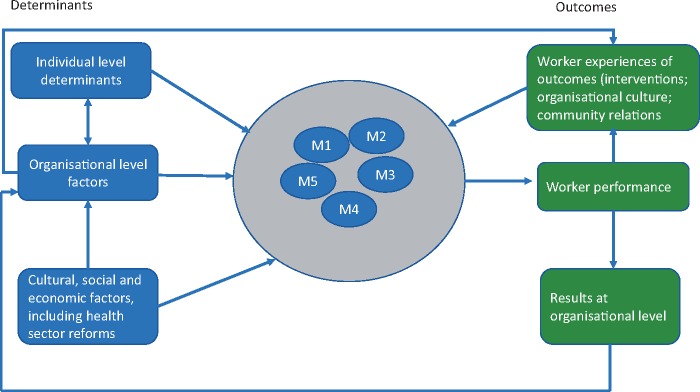
Conceptualizing motivation. This figure is adapted from Franco *et al.* 2002 to convey the determinants and outcomes of motivation and the dimensions of motivation within a multi-dimensional framework. M1–M5 are factors, which represent different dimensions of motivation. Within self-determination theory, these could be the following: M1—motivation factor 1 (e.g. integrated motivation), M2—motivation factor 2 (e.g. identified motivation), M3—motivation factor 3 (e.g. introjected motivation), M4—motivation factor 4 (e.g. external regulation), and M5—motivation factor 5 (amotivation) Tremblay *et al.* 2009.

The measurement of motivation is more difficult. A key question is whether to measure motivation itself (a ‘direct’ measure) by, e.g. seeing how a programme affects intrinsic motivation; or to instead measure the things that affect or are affected by motivation (‘proxies’, ‘indirect’ measures). Direct measures of motivation are typically derived through measurement scales within a survey or through qualitative methods (Inceoglu *et al.* 2012; Deci and Ryan 1985). For example, JL and ED examined whether financial incentives crowd out intrinsic motivation using measurement scales grounded in Self-Determination Theory in health worker surveys in Burkina Faso and Afghanistan (Lohmann *et al.* 2017; Dale 2014) (see Figure 1). Indirect measures can be equally derived through surveys or qualitative methods or through experimental games or observations of behaviour. For instance, the Franco framework, which has been widely used in health worker motivation studies in low- and middle-income countries (LMICs), measures determinants of motivation with a series of psychometric scales in a health worker survey, examining the individual (e.g. self-efficacy, desire for achievement), organizational (e.g. management support, financial rewards), and external level determinants (relations with the community/patients) (see (Franco *et al.* 2002; Mbindyo *et al.* 2009 b; Mutale *et al.* 2013; Morrison *et al.* 2015; Chandler *et al.* 2009). JB and AK used this approach to examine the effects of primary care reforms on motivation composition and levels in Tanzania. Also in Tanzania, Leonard and Masatu (2010) made use of the Hawthorne effect (i.e. performance impact of being observed) to investigate health workers’ intrinsic motivation. In choosing a motivation measure, it is important to consider whether and how a programme is likely to affect motivation and how this would affect worker performance. This paper outlines the steps in measuring health worker motivation with Likert-type psychometric scales, as part of surveys, and in analysing such data, using examples from our respective research and the wider literature.

### Step 2: developing and pre-testing a tool

Having selected a conceptualization of motivation, the first step in developing a survey tool is to identify a set of questions to measure motivation, referred to as a measurement scale (DeVellis 2012; Fowler 2009). If the aim is to understand the composition of motivation, then it is helpful to anticipate potential motivation dimensions with reference to theory and the intervention in question (Prytherch *et al.* 2012). Focus group discussions or in-depth interviews with health workers can also help identify dimensions and ensure appropriate communication of these concepts in the local language (Box 3) (e.g. Agyepong *et al.* 2004; Sacks *et* al. 2015). Once relevant dimensions have been identified, these need to be formulated as questionnaire items (i.e. statements or questions). A good first step is to review existing scales (see Mbaruku *et al.* 2014; Hotchkiss *et al.* 2015; Bonenberger *et al.* 2014; Inceoglu *et al.* 2012 and Supplementary Annex S1) and to decide on positive or negative wording, response scales and the number of response options (Box 4). It is recommended to include a minimum of three items per dimension (Little *et al.* 1999; Guilford 1952), although with a new scale, 4–5 items are recommended as some items may not perform well. To enable subsequent validity checks (Step 5), it is also important to collect data on variables that are expected to be related to motivation or motivation dimensions, such as motivational outcomes, e.g. intention to quit or organizational commitment (Hagopian *et al.* 2009; Bonenberger *et al.* 2014), or measures of the knowledge practice gap (Leonard and Masatu 2010) or other performance measures. It is also important to collect data on variables which might influence provider responses to items (health worker or facility level characteristics) (Chandler *et al.* 2009; Mbaruku *et al.* 2014; Hotchkiss *et al.* 2015; Franco *et al.* 2004). Researchers also need to decide on the mode of survey administration (see Box 5). As with all surveys, it is recommended to pre-test the motivation measurement tool with a small sample of health workers (see Prytherch *et al.* 2012) and to proceed through Steps 4 and 5.

Box 3. Finding the right terms

Focus group discussions and interviews are an important way of identifying the appropriate way of communicating concepts related to motivation and eliciting meaningful responses in local language. Constructs such as motivation are not easily and directly translated and understood in the same way across cultures. In Afghanistan, two focus group discussions with seven health workers in two different facilities in Kabul were conducted exploring general questions on reasons for choosing the profession and attitudes towards work. We realized through this process that the word most frequently used by respondents in these discussions when referring to motivation in Dari was the word for encouragement, tashweeq. In Dari, tashweeq means ‘evocation of shauq,’ shauq being a word for desire, zeal, or inclination. When it is combined with the verb ‘kardan’ (to do), tashweeq kardan, it means to encourage. However, when combined with the verb ‘shodan’ (to become), tashweeq shodan, it might acquire an intrinsic aspect. However, in Persian, the word ‘angize’ means motivation. However, ‘angize’ was used more rarely, although it seemed to be understood by all health workers (Dale 2014). This is very similar to findings of the qualitative study in Kenya and Benin (Mathauer, Imhoff, 2006), according to which over 50% of health workers in Benin equated motivation with prospective "encouragement" with one fourth of these explicitly mentioning financial encouragement, while another 40% considered "being motivated" as having the necessary means to work and get recognition. As the authors of this study put it, the majority understood motivation as ‘an incentive, and not as a state of mind’ (Mathauer, Imhoff, 2006). In Tanzania, respondents differentiated between: motivation as a desire to serve ‘kuwa na moyo’*;* and motivation driven by monetary benefits, social recognition, power ‘motisha’.

This type of qualitative analysis through focus group discussions with the target group will play an important role in refining one’s conceptualization of motivation, developing items for the questionnaire, and finding a way of eliciting the appropriate construct. This will be context specific and may need to be adapted to different respondent types, particularly if there are large differences in the level of education (e.g. doctor versus community health worker).

Box 4. Question format

There are different ways of structuring questions in motivation surveys. When assessing motivational composition, researchers typically use a series of items, against which respondents have to rate their level of agreement. These can be phrased as affirmations or in the negative. For example when examining the relevance of staffing to motivation researchers could use the following item: ‘there are enough providers at this facility’. This could be phrased using negative wording: ‘there are not enough providers at this facility’; or could be presented in the affirmative, but conveying a negative concept: ‘there are insufficient providers at this facility’. The use of negative wording, or negative concepts in the affirmative may help to reduce acquiescence bias (Prytherch *et al.* 2012) but can sometimes confuse respondents, and their responses to equivalent positive items do not always correspond. In Burkina Faso, for instance, JL presented respondents with the following two items (spaced, with other items in between):

1. ‘I wish I worked in another health facility’ and

2. ‘I wouldn’t want to work in another health facility’.

Although both measure health worker attitudes towards work at their facility, the following means were obtained on a 0–10 response scale: 1) 5.2; 2) 3.0 (non-reversed). Respondents’ answers to the two questions correlated at only -.24.

Items should be kept simple, avoiding leading or double-barreled questions. They can be assessed for readability using the Flesch reading ease formula using Microsoft Word. It is recommended that items should not exceed the reading level of 6th–7th grade. It is also recommended to check understanding among a representative sample of health workers, as education levels vary by setting (Dale 2014).

Along with the development of items, researchers must also choose a response scale. The responses to items can take many forms (Streiner *et al.* 2008). Options include dichotomous response options (e.g. yes-no, true–false, agree–disagree) or rating scales. The Likert rating scale is widely used in the literature on health worker motivation (e.g. Chandler *et al.* 2009; Weldegebriel *et al.* 2016;Alhassan *et al.* 2013), and preferred by the authors of this paper. Considerations here include the number of response categories, and whether or not to include a neutral category. Too few categories will result in a loss of information and less variance in data, impacting reliability. A larger number of categories will make the tool more sensitive to detecting a shift in motivation levels if that is a research aim. However, too many categories might overwhelm respondents and put into question meaningfulness of differences between categories. The number of categories should be commensurate with respondent ability to discriminate which will vary by context and numeracy (Preston and Colman 2000). Five to nine categories are considered in most circumstances (Streiner *et al.* 2008). In Afghanistan, the team used five categories (Dale 2014). In Tanzania, the team opted for four response categories as respondents had difficulty processing more than that. In Burkina Faso, the team used 11 categories with a visual aid (depicting cubes as a visual representation of the ‘amount’ of agreement) as a compromise between the team’s need for sufficient variance in data, respondents’ processing capacity, and local ‘positivity norms’ leading respondents to primarily consider the positive end of the response scale regardless of their actual sentiments. However, the 11 categories might have been overwhelming to respondents, so the team would recommend seven to nine categories in future research (Lohmann *et al.* 2017).

A number of response options can be associated with the Likert scale for motivation measurement. ‘Agreement-disagreement’ response options are often used when measuring motivation through proxies. Response options relating to the ‘importance’ or the ‘frequency’ of a given item may also be appropriate in certain contexts. It is important to ensure the response options are consistent with the associated items. For example, when enquiring about the work environment, researchers could present the question as follows, using a 4 point Likert scale: *This facility is well stocked with drugs and supplies.Strongly agree, agree, disagree, strongly disagree.*

Items and response options should be chosen so as to be meaningful in a given context. In Afghanistan, the team presented items such as ‘I work in this job because I have a chance to help other people through my work’, asking respondents to indicate their degree of agreement (Dale 2014). The research in Burkina Faso found respondents to have difficulties with the abstraction processes required to answer such items, and instead presented a list of reasons why people might be motivated to work in their job, asking respondents to indicate to what extent these were important to them personally (Lohmann *et al.* 2017). It is not a problem to include different response options within the same questionnaire. However, it is advisable to use the same response format (both in terms of number of categories and response options) for a set of items pertaining to the same construct (i.e. if intrinsic motivation is measured with 5 items) so that comparability of responses to items measuring the same construct is preserved. The advantage of response options that relate to ‘satisfaction’ rather than ‘agreement’ is that they can be communicated in conversational language, and do not present the respondent with a pre-formulated response (which may be leading). Though this formulation would not be appropriate for direct measures of motivation.

Box 5. Survey administration

Self-administered surveys are often seen as preferable as they maximize perceived confidentiality, thus minimizing social desirability biases, and allow respondents to choose the time of response. However, they are prone to misunderstanding of instructions and acquiescence bias (‘rushing through’ e.g. by always answering ‘yes’ or giving the highest/lowest score), and tend to have relatively low response rates without substantial follow-up efforts by the researcher. They also require participants to be able to read and write, which is usually not a concern with skilled healthcare personnel, but can be with auxiliary personnel or community health workers.

For these reasons, interviewer-administered face-to-face surveys are much more common in public health research in LMICs. The risk for social desirability bias in face-to-face surveys can be minimized by training interviewers to be sensitive to the private nature of interview questions, by repeatedly reminding respondents of the confidentiality of their answers, and by administering the survey in a setting that maximises privacy.

In Burkina Faso, JL opted for a ‘hybrid approach’ to combine the advantages of both forms of administration, minimizing the risk of response biases while at the same time maximizing response rates and data quality. Interviewers read out instructions and all items as in a face-to-face interview. However, respondents were given a separate questionnaire copy to read along and enter their own answers privately rather than disclosing them to the interviewer (Lohmann *et al.* 2017).

### Step 3: sample size considerations and sampling

Sample size is a further consideration prior to survey administration. The techniques used to assess the validity of the motivation measure (Step 5) require certain minimum sample sizes, dependent on the number of dimensions, items and other factors (Kline 2010). Commonly used rules of thumb for factor analytical techniques are ‘no less than100 observations’ (Gorsuch 1983; Kline 1979; Kline 2010; MacCallum *et al.* 1999), with 50 observations often considered the absolute minimum (de Winter *et al.* 2009) for exploratory factor analysis; and 200 observations the minimum for confirmatory factor analysis (DeCoster 1998). If sub-group analysis is planned (e.g. comparing motivation between different cadres of health worker), these sample sizes should be achieved for each sub group. As in any other study, sample size requirements also depend on the planned substantive analyses (Step 7). The sample size requirements to analyse motivation determinants depends on the type of model used, with a standard linear regression model having lower sample size requirements than structural equation models (Wolf EJ et al. 2013). When considering the impact of a programme on motivation, power considerations are also important, but estimations of effect size tend to be difficult given that they are highly dependent on the motivation measure itself. Often, motivation surveys are administered to health workers who are present on the day of the facility visit. These health workers are likely more motivated than their counterparts who are not present at facilities, and it would be important, where possible, to also make provisions to interview health workers who are absent from facilities on the day of the visit.

### Step 4: exploratory data analysis

Once the data have been collected, it is important to start with an exploration of the data, estimating mean and median scores and distributions for each item, and checking for missing data. The empirical literature on health worker motivation has tended to analyse Likert responses as continuous variables, given that the underlying motivation construct is assumed to be continuous. However, there has been some debate as to whether this approach is appropriate given the ordered categorical nature of Likert scales, though there is evidence it may make little difference in practice—see (Carifio and Perla 2008; 2007), for more discussion of this point.

A high level of missing values may indicate that an item was not well understood by respondents (Raykov and Marcoulides 2011; Little 1992). Where missing values exceed 10% researchers should weigh the option of dropping the item against maintaining measurement consistency across respondents. It is generally recommended to consider dropping items where >80% of respondents provide the same answer to a question as such items have little discriminatory value (Streiner *et al.* 2008). Direction of response for each item, particularly for those that were negatively worded, should also be checked for plausibility.

### Step 5: assessing validity of motivation measures

Before using motivation measurements in core analyses, researchers should ensure the measures are valid or that they measured what was intended (DeVellis 2012; Fowler 2009).

If motivation is considered to be multidimensional, the first step in validating the measure is to determine the composition of motivation, or the underlying dimensions, to confirm or modify initial hypotheses. This is typically done with factor analytical techniques; either exploratory or confirmatory (see Box 6). Before doing so, it is important to check the factorability (or reducibility) of the data (e.g. inspect item correlation matrix; Bartlett test of sphericity test; Kaiser–Meyer–Olkin test (Yong and Pearce 2013)).

Box 6. What is the difference between PCA and factor analysis?

Although often used interchangeably, PCA and factor analysis are conceptually rather different. PCA is primarily a data reduction technique used to create indices (or reduce a number of variables into a single index). Factor analysis is an umbrella term referring to different techniques aiming to relate the underlying unobservable construct(s) (‘latent variable’) to be measured, i.e. a respondent’s level of motivation, to the items intended to measure it. Two general types of factor analysis are distinguished: exploratory factor analysis (EFA), where there is no a priori theory about which items measure which factor and the researcher derives the ‘factor structure’ (number of factors, e.g. different motivation dimensions; item-factor assignment) of the scale from the data; and confirmatory factor analysis (CFA), where the researcher has a priori hypotheses of the scale’s factor structure and examines whether these hypotheses are consistent with the data. Within EFA there are two main statistical methods for factor extraction: principle axis factoring (PAF) (the default method used by Stata when using the ‘factor’ command) and principal component analysis (PCA, with rotation) (Costello and Osborne 2005). Structural equation modelling is the standard statistical technique for CFA and can also be used for EFA. However, alternative data extraction methods exist for both types of factor analysis and might be more appropriate depending on the data (Costello and Osborne 2005).

#### Confirmatory factor analysis

Confirmatory factor analysis (CFA) is used where the researcher has strong assumptions regarding the dimensionality of the scale from prior qualitative research, theory, or prior use of the scale (Brown 2006). Researchers must specify the number of dimensions (or ‘factors’) and which items measure which dimension or factor. For example, the researcher may have pre-identified three motivation dimensions: ‘work environment, salary, and conscientiousness’; and clearly assigned to each a number of items (e.g. the item ‘availability of drugs’ may be associated with the dimension ‘work environment’).

CFA results indicate the extent to which the pre-specified dimensions are reflected in the data. Good model fit confirms that the dimensions are relevant and can be readily interpreted. Several statistical approaches can be used to confirm whether the dimensions are relevant using CFA, with structural equation modelling being the most common (Kline 2010). If health workers were sampled from facilities, it is important to account for the clustered nature of the data in the analyses described subsequently (Supplementary Annex S2). In the absence of good model fit, modifications may be made by, e.g. removing or reassigning items, or modifying the choice of dimensions. Careful consideration of the implications of eventual modifications for the underlying conceptualization of motivation is recommended.

In many studies of health workers in LMICs, there has been limited if any prior study of motivation meaning that it is unclear what the underlying dimensions or factors might be. For this reason, exploratory factor analysis (EFA) has been most widely used in these settings.

#### Exploratory factor analysis

When constructing new scales and/or applying them to novel contexts, researchers are often not entirely sure how many and which motivation dimensions the scale items measure. Unlike CFA, EFA does not impose any a priori assumptions on the number of motivation factors, and the assignment of items to factors. Rather, EFA is used to identify meaningful dimensions of motivation, and to determine which items measure which dimension, on the basis of respondents’ answer patterns to the scale items. EFA is sometimes used to generate a theory about the relevant dimensions of motivation that are then used in a CFA. With sufficient sample size, EFA can be performed on one part of the data, and the generalizability of the extracted factors can be determined using CFA on the other (Raykov and Marcoulides 2011).

##### Factor extraction

A variety of statistical approaches can be used to extract factors using EFA. Principal component analysis (PCA) and principal axis factoring (PAF) are the most common (Williams *et al.* 2012; DeVellis 2012). Rotation is used to simplify and clarify the results of EFA facilitating the identification of factors. There are two main types of rotation: orthogonal and oblique, with the main difference being that the latter allows for some correlation between factors whereas the former does not. The former has been widely used because it is believed to be simpler (e.g. Chandler *et al.* 2009). However, as motivation dimensions are unlikely to be unrelated (e.g. there will be some association between different factors, such as drug availability and supervision or management involvement in facilities), the latter approach is preferable.

##### Deciding how many factors and which items to retain

**Figure 2.**
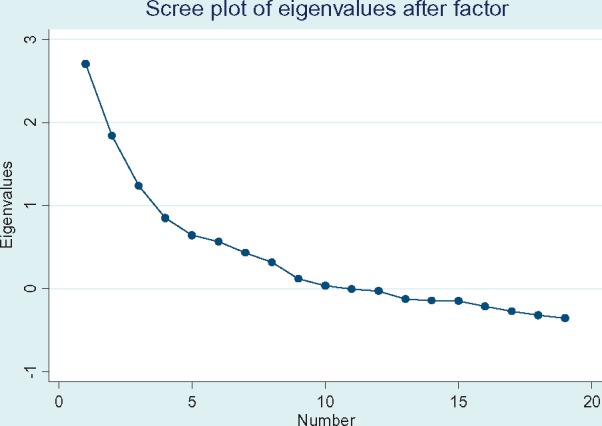
Scree plot for survey data collected in Tanzania. Based on visual inspection alone, 5 factors appear to be the turning point after which the plot levels off (though it does so again at 8). However, using the Kaiser criterion (retaining factors with an eigen value of 1 or more), 3 factors would be retained

The full list of factors resulting from an unrestricted EFA will correspond to the number of items included. The researcher must decide how many to retain. This decision will be based in part on theoretical considerations: *how many dimensions is it reasonable to expect?* and whether the resulting factors can be readily named and described. The following can also help determine the number of factors: a common rule of thumb is to retain factors that have eigenvalues over 1 (the Kaiser criterion) (Hayton *et al.* 2004; Kaiser 1960); visually examine eigenvalue plots for the natural bend or break point in the data where the curve flattens out (Figure 2) (Cattell 1966; Chandler *et al.* 2009); examine the total variance explained (aim to explain 50–75% with the least number of factors). In practice if using the ‘factor’ command in Stata, there are different cut-off values for factor retention that are built into the software depending on the method of factor extraction selected.^1^ We encourage researchers to think critically about how many factors make sense in their context rather than to blindly accept these arbitrary cut-offs.

It is also important to examine the factor loadings for each item. In EFA, all items will load on all factors to some degree. The aim is to determine which items are most indicative of which factors, based on the degree of factor loading, with 0.3 (Tabachnick and Fidell 2007) and 0.4 being commonly used (Chandler *et al.* 2009) as cut-off values for ‘substantive loadings’. Higher thresholds are recommended for small sample sizes. The ideal scenario is that each item has a substantive loading on only one factor and is conceptually close to the other items with substantive loadings on that factor. However, this is often not the case, and researchers will have to decide whether for instance to define a different number of factors or to eliminate items with low factor loadings. EFA is invariably an iterative process, as results change with the number of factors retained and items included.

##### Interpreting and naming factors

When interpreting and naming factors, it is important to refer back to the exact wording of the scale items and the aspects of motivation they were designed to measure. Often, the interpretation of a factor is relatively straightforward from the items loading on it. For example, in Tanzania, the following three items: availability of drugs, supplies and equipment at the facility, had substantive loadings on the same factor. All three clearly pertained to the ‘work environment’. It is possible that some items may not fit semantically with the factor they load on. For example, in the same Tanzanian study, 5 items loaded substantively on another factor. Four of the items were related to ‘management and supervision at the facility’, but one item did not appear to fit with that definition: ‘relationship with local leaders in the community’. One explanation for such cases is a divergence between respondent and researcher interpretation of an item. In this case, respondents may have considered community leaders together with managers given their joint involvement in facility governing committees. Interviews/focus groups can be used to shed light on respondent understanding and if the item is found to be related to the dimension it can be retained. Another reason for ‘lone items’ is that they are related to a sub-dimension of motivation that did not emerge as a separate multi-item factor simply because the scale contained only one item pertaining to it. In such cases, researchers must decide whether to keep the item as a (psychometrically suboptimal) single-item measure, or whether to drop it. In some cases, clusters of items that do not fit well together may be a statistical artefact: EFA groups items based on response patterns without considering how these items relate to each other semantically. The idea is that people respond similarly to items of similar content, because these items tap into the same construct. However, this may not be the case. For instance, a person might feel equally motivated by intrinsic and extrinsic factors and thus assign similar numeric values to related items. In an EFA, we might then end up with a one-factor solution combining extrinsic and intrinsic motivation items. However, this does not mean that extrinsic and intrinsic motivation is the same. Therefore, it is important to interpret factor analysis results together with theory and knowledge of the context.

### Step 6: measurement reliability

Reliability refers to the extent to which the measurement scale produces similar results under similar conditions (DeVellis 2012). Internal consistency based on Cronbach’s α coefficient, the average correlation between items, is the most widely used statistic to assess measurement reliability. In recent years, however, psychometricians have cautioned against the use of α, for conceptual reasons (Yang and Green 2011) and due to its vulnerability to outliers, non-normal data, small number of items, and low variability in total scores (Greer *et al.* 2006; Cortina 1993; Sijtsma 2009; Cronbach and Shavelson 2004). Factor-analysis-based estimation of reliability is now preferred to Chronbach's alpha (Yang and Green 2011; Raykov and Marcoulides 2011). When estimating Chronbach's alpha, it is recommended to use the polychoric correlation matrix instead of a Pearson correlation matrix (Gadermann *et al.* 2012; Dale 2014). For multidimensional measures of motivation, Chronbach's alpha should be estimated for each dimension (Cortina 1993). A typical recommended cut-off level for α has been 0.70, however, as this parameter depends on the number of items among other things, this value should be treated cautiously.

Test–retest measures the degree to which health workers would provide the same responses to items in a repeat survey. In public health studies where the scale development is not the central focus, test–retest validation studies are unfortunately often not feasible for practical reasons. If a retest is possible, it is important to choose the time delay between test and retest in a way that the underlying construct measured with the scale can be assumed to have remained stable.

When motivation is to be compared across different subgroups (e.g. women vs men, doctors vs other cadres, different language groups, across countries), the scale should be tested for equal measurement properties across subgroups. If measurement invariance is not established, there is a risk that subgroup differences are not due to differences in motivation but to differences in the performance of the measure in the subgroups (Vandenberg and Lance 2000). Measurement invariance testing is usually done in a CFA framework (see Supplementary Annex S3).

### Step 7: core analysis

Once validity and reliability are established, the motivation measure can be used within analysis, depending on the objective of the study. If the objective is to describe motivation levels, item responses can be combined into a composite score, typically calculated as the arithmetic mean of health worker responses. Means can be calculated either as unweighted means, i.e. all items have the same weight, or one can give more weight to some items than to others, which may be preferable if the EFA and/or CFA results show substantially different factor loadings between items. In such cases, the loadings can serve as weights. If the motivation measure was found to be multidimensional at the EFA/CFA stage, scores are calculated separately for each dimension. Researchers have sometimes also estimated an overall motivation score by combining item scores across dimensions, e.g. Gow *et al.* 2013; Bhatnagar and George 2016; Hagopian *et al.* 2009; Mbindyo *et al.* 2009 b. Where dimensions are deemed conceptually distinct, such practice makes limited sense and risks evening out important differences across dimensions. If researchers wish to capture overall motivation, a related item can be included in the measurement scale (e.g. ‘Overall, how motivated do you feel?’).

If the objective is to understand determinants or consequences of motivation, or changes in motivation over time, there are two main analytical options: using composite scores (‘manifest variables’), or using a latent variable approach where the relationship between motivation and other variables of interest are inferred directly from the scale items, without the estimation of composite scores.

Composite scores can be used as predictor or outcome variables in a regression model. However, much of the variance contained in the individual items is ‘averaged out’ by the calculation of a mean composite score (Borsboom 2006; Skrondal and Laake 2001).

With a latent variable approach in SEM, associations between motivation and other variables of interest are directly estimated from the items via the latent variable/s. This approach provides more accurate estimates of the relationship between motivation and other variables as all information contained in the dataset is preserved. However, large structural equation models are complex and difficult to handle and have large sample size requirements. 

### Step 8: presenting findings

**Figure 3.**
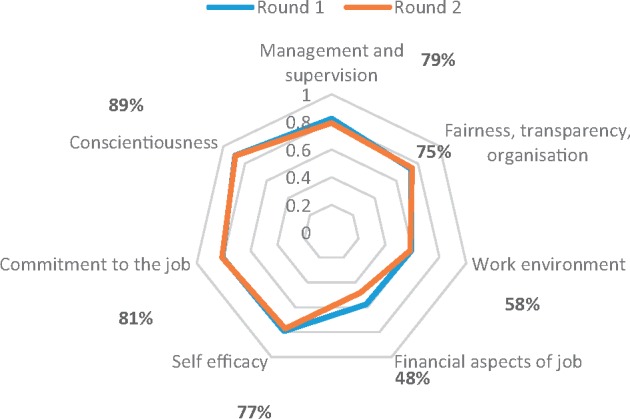
Spider diagram showing changes in composite scores over time. In Tanzania, management and supervision, fairness, transparency, organisation, the work environment, financial aspects of the job, and intrinsic factors (commitment, conscientiousness and self-efficacy) were identified as potential dimensions of motivation. Conscientiousness, commitment to the job, and management and supervision scored highest.

**Figure 4.**
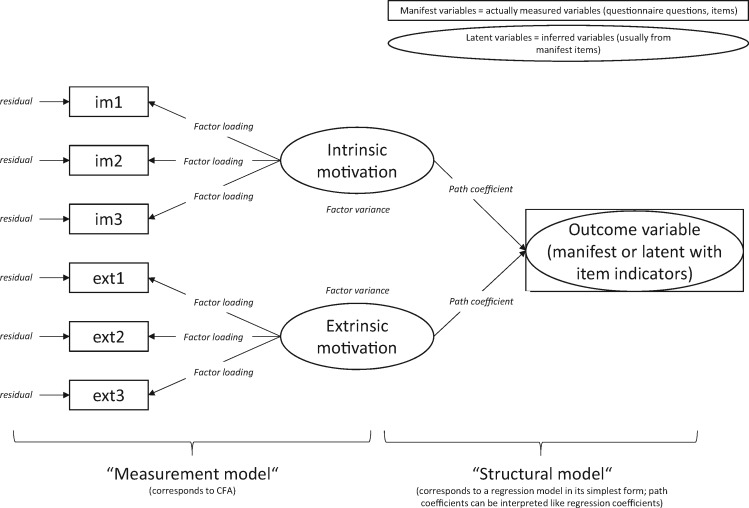
Illustration of the use of structural equation models for confirmatory factor analysis and the analysis of motivation determinants

When reporting findings, it is important to be transparent as to the steps taken to generate results and decisions made during this process. It is standard practice to present all items used to measure motivation along with their mean scores and standard deviations. Results for EFA and/or CFA should be reported, including factor loadings for each item and model fit. If composite scores are calculated, mean scores and standard deviations should be reported. Spider diagrams or other graphs can be helpful to visualize composite scores and variance across dimensions and changes over time (Figure 3). If SEM is used, a visualization of the model including parameter estimates can be informative in addition to model fit information (Figure 4).

## Discussion

We have highlighted the steps involved in measuring and analysing health worker motivation survey data and the importance of having a clear conceptualization of motivation as a single or multi-dimensional construct, prior to undertaking measurement.

We have described the use of exploratory or confirmatory factor analysis to identify or confirm motivation dimensions. Most of the existing health worker motivation literature in LMICs uses EFA (Alhassan *et al.* 2013; Mbindyo *et al* 2009 b; Bonenberger *et al.* 2014; Chandler *et al.* 2009). There is potential for greater use of CFA, especially in studies that have clearly articulated dimensions of motivation, based on theory or prior formative research (e.g. Weldegebriel *et al.* 2016; Agyepong *et al.* 2004; Ojakaa *et al.* 2014; Franco *et al.* 2004; Hotchkiss *et al.* 2015). Some studies had pre-defined motivation dimensions and presented a descriptive assessment of item scores and means across dimensions without employing factor analysis to validate these results (e.g. Ojakaa *et al.* 2014; Ssengooba *et al.* 2007; Dieleman *et al.* 2006; Lephoko *et al.* 2006). While descriptive analysis is an important first step in any motivation study, it is difficult to definitively assess how well items measured each dimension, and whether assumptions about composition were accurate, without doing factor analysis. However, as tools become more widely used and validated in different contexts and languages, and our knowledge of motivation dimensions in these contexts grows, factor analysis may not always be required.

Much of the empirical research has been aimed at identifying the composition of motivation and factors driving motivation, looking at how these vary between groups and over time in response to policy change. In such cases, the focus is on the relative differences/changes over time/between groups rather than absolute levels. We have shown how composite scores can be calculated if the interest is in absolute motivation levels at a certain point in time. However, researchers should be cautious in the interpretation of these scores. Responses to questions about motivation may be affected by social desirability bias. For example, respondents may provide high scores on intrinsic motivators, items relating to commitment, punctuality, or attitude to work, regardless of how they really feel. This may not mean they really are highly intrinsically motivated, but might be a result of them ‘anchoring’ their responses differently for different dimensions and items. Careful design of the scale can shed light on such issues and inform interpretation. Most published studies have used qualitative methods to inform the design of the scale (Sacks *et al.* 2015) and/or as part of the research study (Chandler *et al.* 2009) to maximize content validity and facilitate an accurate interpretation of findings. Checking that associations between motivation measures and motivational outcomes and/or health worker characteristics conform to expectations is also important. A number of studies have examined and reported determinants of motivation to assess construct validity (e.g. Hotchkiss *et al.* 2015; Franco *et al.* 2004); however, this is not done systematically. Some studies have also examined the relationship between motivation and turnover intentions and performance outcomes in health workers in low and middle income settings (Bonenberger *et al.* 2014; Alhassan *et al.* 2013). More extensive empirical research has examined this question in relation to other types of workers in high income settings (Deci *et al.* 2017). More research of this kind is needed in LMIC settings, in order to assess the validity of the motivational measure and also to understand the extent to which motivation acts as a mediator of better performance in different contexts and in response to different interventions.

As increasing efforts are made to improve the performance of health workers to provide more effective care in LMICS, researcher and policy interest in measuring and understanding motivation in surveys is likely to remain high. We hope this paper provides a useful introduction for those wanting to gain a better understanding of the methodology and the process of designing surveys to measure motivation in LMICs and the methods used to analyse and interpret their findings.

## Supplementary Data

Supplementary data are available at *Health Policy and Planning* online.

## Supplementary Material

Supplementary Data

## Footnotes

1 For instance, for principal axis factor, it’s all factors with eigenvalues greater zero. For PCA, it’s all factors with eigenvalues greater than 1 (the Kaiser criterion).
