# Supplementary material for: How to do (or not to do)… Measuring health worker motivation in surveys in low- and middle-income countries
Source: Health Policy Plan. 2017 Nov 20;33(2):192–203. doi: 10.1093/heapol/czx153 (PMC5886192; doi:10.1093/heapol/czx153)
Supplement: Supplementary Data [file czx153_supplementary_web_annexes_edit_final.docx]

**Supplementary Web Annexes**

**Title: How to do (or not to do)… Measuring health worker motivation in surveys in Low and Middle Income Countries**

**ANNEX 1: Review of available tools for measuring health worker motivation in surveys**

The most widely used tool to measure health worker motivation in LMICs is that developed and explained in (Bennett et al. 2001), which were first applied in Georgia and Jordan. Table 2 in (Franco et al. 2004) shows the survey tools that were used to inform that tool presented in Bennett. The tool has since been adapted for use and validated in Kenya at hospital level (Mbindyo et al. 2009), in Zambia (Mutale et al. 2013); and for use among auxiliary nurse midwives in Nepal (Morrison et al. 2015), and among primary care workers in Tanzania, South Africa and Malawi (Blaauw et al. 2013). A tool developed and applied to primary care providers in Tanzania, Ghana and South Africa, is set out in Pyrech et al. including a commentary on the performance of each of the items contained (Prytherch et al. 2012).

The Healthcare Provider Work Index (Aiken et al. 1997), a tool developed for use in the US to examine nurse motivation, was adapted for use used in Malawi (McAuliffe et al. 2009).

Edward L. Deci and Richard M. Ryan, authors of the Self-Determination Theory (SDT), have published a collection of SDT-based tools, which are publically available at <http://selfdeterminationtheory.org/>. These tools were used and adapted for the studies in Afghanistan (Dale 2014) and Burkina Faso (Lohmann et al. 2017).

**Annex 2: Dealing with Clustering**

While clustering is always taken into consideration in the analysis of household survey data, often this is not done within the analysis of health worker motivation data, although health workers are typically sampled from facilities, and are clustered at this level. We recommend that researchers adjust for clustering at the facility level within their analysis where appropriate as would be done routinely in other studies. In the Afghanistan study carried out by ED, standard error computations used a sandwich estimator in order to account for non-independence of observations due to cluster sampling at the facility level (Muthén & Muthén 2012) (Dale 2014).

**ANNEX 3: Measurement invariance testing in detail**

Measurement invariance testing is done for CFA models after good model fit has been demonstrated for the overall sample (2.1). Conceptually, invariance testing is done by simultaneously estimating the model in all subgroups, and by making it gradually more equal by fixing parameters such as factor loadings to equality in the subgroups. In three steps, more and more parameter constraints are introduced. If model fit does not become significantly worse in relation to the next less constrained model, the motivation scale can be assumed to measure at same in all subgroups.

| **Test for** | **Interpretation** | **Model constraints** |
| --- | --- | --- |
| Configural invariance | Tests for the assumption of the same underlying factor structure in all subgroups, i.e. whether the overall model fits similarly well in all subgroups  (e.g., does the scale measure the same five motivation factors in Dari and Pashtu?) | No specific constraints are imposed on the estimated parameters. |
| Metric invariance | Tests whether the same constructs are measured across subgroups, i.e. whether respondents in different subgroups attribute the same meaning to the respective factors (e.g., are the five factors interpreted in the same way in Dari and Pashtu?) | - Factor loadings estimated freely, but constrained to equality in the subgroups |
| Scalar invariance | Tests whether subgroups can be compared on their mean scores, or if subgroups score systematically different (at same level of underlying factor) for certain items (e.g., at the same underlying level of intrinsic motivation, do Dari respondents score the same as Pashtu respondents?) | - Factor loadings estimated freely, but constrained to equality in the subgroups - Item intercepts estimated freely, but constrained to equality in the subgroups |
| Residual variance invariance | Tests whether the proportion of contamination by other constructs as measured by the different items (i.e. variance that is not explained by the intended factors) is equal across groups, and whether measurements are thus fully comparable across groups (e.g., is item 1, intending to measure intrinsic motivation, contaminated by other constructs to the same extent in Dari and Pashtu?) | - Factor loadings estimated freely, but constrained to equality in the subgroups - Item intercepts estimated freely, but constrained to equality in the subgroups - Item residual variances estimated freely, but constrained to equality in subgroups |

For a description of how to do measurement invariance testing in Stata, see the help file for *estat ginvariant* in the Stata Manual (Stata.) and (Gregorich). For a description in Mplus, see (Hoffman) and the references listed here.

**References**

Aiken LH, Sochalski J, Lake ET. 1997. Studying outcomes of organisational change in health services. *Medical Care.*, **35**: NS6-NS18.

Bennett SFM, Kanfer R, Stubblebine P. 2001. Major Applied Research 5: Technical Paper 2: The Development of Tools to Measure the Determinants and Consequences of Health Worker Motivation in Developing Countries. . Abt Associates Inc.

Blaauw D, Ditlopo P, Maseko F, et al. 2013. Comparing the job satisfaction and intention to leave of different categories of health workers in Tanzania, Malawi, and South Africa. *Glob Health Action*, **6**: 19287.

Brody CM, Bellows N, Campbell M, Potts M. 2013. The impact of vouchers on the use and quality of health care in developing countries: A systematic review. . *Glob Public Health.*: 26.

Dale E. 2014. Performance based payments, provider motivation and quality of care in Afghanistan. John Hopkins University.

Franco LM, Bennett S, Kanfer R, Stubblebine P. 2004. Determinants and consequences of health worker motivation in hospitals in Jordan and Georgia. *Soc Sci Med*, **58**: 343-55.

Gregorich SE. Stata Paper Examples: Do self-report instruments allow meaningful comparisons across diverse population groups? Testing measurement invariance using the confirmatory factor analysis framework. <http://www.ats.ucla.edu/stat/stata/paperexamples/gregorich/:> Institute for Digital Research and Education, UCLA.,.

Hoffman L. Multiple Group CFA Invariance Example (data from Brown Chapter 7) using MLR Mplus 7.11: Major Depression Criteria across Men and Women (n = 345 each). <http://www.lesahoffman.com/948/948_Example9a_CFA_Multiple_Group_Invariance.pdf>.

Lohmann J, Souares A, Tiendrebéogo J, et al. 2017. Measuring health workers’ motivation composition: validation of a scale based on Self-Determination Theory in Burkina Faso. *Hum Resour Health*, **15**: 33.

Mbindyo PM, Blaauw D, Gilson L, English M. 2009. Developing a tool to measure health worker motivation in district hospitals in Kenya. *Hum Resour Health*, **7**: 40.

McAuliffe E, , , Bowie C, Manafa O, et al. 2009. Measuring and managing the work environment of the mid-level provider--the neglected human resource. *Hum Resour Health.* , **7**.

Morrison J, Batura N, Thapa R, Basnyat R, Skordis-Worrall J. 2015. Validating a tool to measure auxiliary nurse midwife and nurse motivation in rural Nepal. *Human Resources for Health* **13**.

Mutale W, Ayles H, Bond V, Mwanamwenge MT, Balabanova D. 2013. Measuring health workers' motivation in rural health facilities: baseline results from three study districts in Zambia. *Hum Resour Health*, **11**: 8.

Muthén LK, Muthén BO. 2012. *Mplus User's Guide.* Seventh Edition edn. Muthén & Muthén, Los Angeles, CA.

Prytherch H, Leshabari MT, Wiskow C, et al. 2012. The challenges of developing an instrument to assess health provider motivation at primary care level in rural Burkina Faso, Ghana and Tanzania. *Glob Health Action*, **5**: 1-18.

Stata. estat ginvariant — Tests for invariance of parameters across groups. <http://www.stata.com/manuals13/semestatginvariant.pdf#semestatginvariant>
